# Complete chloroplast genome of the federally endangered Menzies’ wallflower *Erysimum menziesii* (Brassicaceae, Anthophyta) from California, USA

**DOI:** 10.1128/mra.01298-25

**Published:** 2026-02-10

**Authors:** Alondra G. Aguilar, Pilar Arreguin, Isabel Bejar, Alejandra Castro, Millo D. Chavarria, Oscar Chavez, Jacqueline Cruz-Martinez, Ruby J. De la Cruz-Ortiz, Paloma L. Degrandis-Figueroa, Kaleigh Gonzalez, Mayra E. Gutierrez-Vargas, Mason Hill, Jeffery R. Hughey, Cameron M. Jones, Kayla Kennamore, Vanessa Lopez, Madry M. Nelson, Itzel Lopez Magana, Jasmine Magana-Zavala, Brianna S. Martinez, Vanessa Mendez Gutierrez, Brayan Mendoza, Jorge E. Morales, Sarahi Ochoa, Rosalie Perea-Lopez, Judith Perez-Rizo, Dalyla Quiroz-Contreras, Emily C. Ramirez, Fidel Ramirez, Izes Ramirez, Tatiana Rozin, Maria G. Salazar, Paulina Saldana-Landeros, Joel Sosa, Joana Vega-Valencia, Andrea Villanueva, Ruby Villicana, Janet Zambrano

**Affiliations:** 1 Division of Mathematics, Science, and Engineering, Hartnell College 17023, Salinas, California, USA; 2 California State Polytechnic University, Humboldt, California, USA; 3 Department of Chemistry, University of Washington 214856, Seattle, Washington, USA; University of Maryland School of Medicine, Baltimore, Maryland, USA

**Keywords:** bioinformatics, conservation genetics, high-throughput sequencing

## Abstract

We present the complete chloroplast genome of the federally endangered plant species *Erysimum menziesii* from Pacific Grove, California, USA. The chloroplast genome is circular, AT skewed (63.4%), and 154,668 bp in length. The genome is quadripartite in structure and contains 85 protein-coding, 37 transfer RNA, and 8 ribosomal RNA genes.

## ANNOUNCEMENT

**Fig 1:**
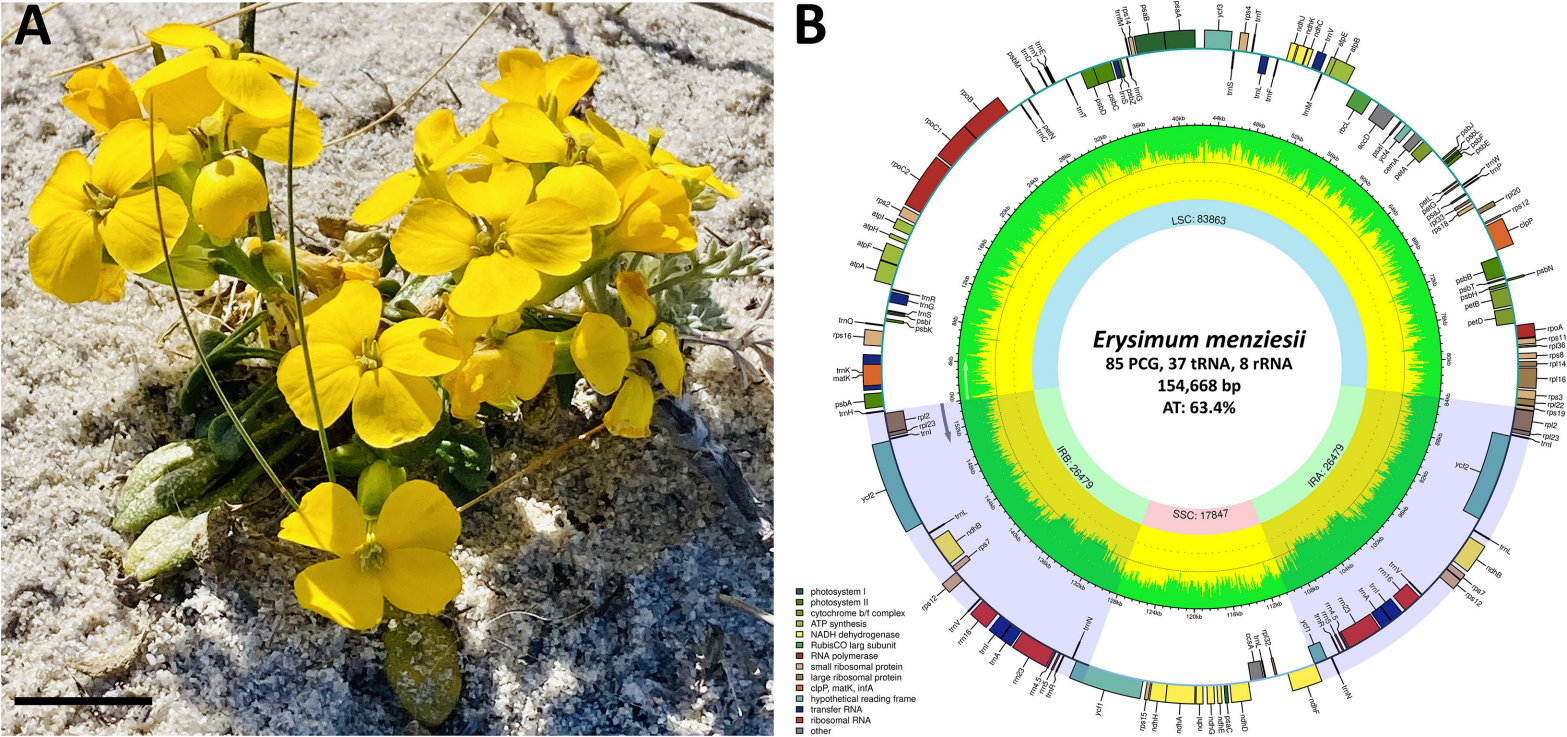
*Erysimum menziesii* habit-inflorescence (**A**) and the complete chloroplast genome map (**B**). (**A**) Specimen of *E. menziesii* from Asilomar, Pacific Grove, California, USA. Scale bar = 2.5 cm. (**B**) Chloroplast genome map of *E. menziesii*. PCG, protein-coding gene. The genome was mapped with Chloroplot 0.2.4 (11). The innermost ring displays the AT content and the direction of transcription, as indicated by the arrows. The final ring displays the genes. Genes transcribed clockwise are on the inside, while counterclockwise transcriptions are positioned on the outside. The color coding corresponds to genes of different groups as listed in the key in the bottom left.

*Erysimum menziesii* (Hook.) Wettst., Menzies’ wallflower (Fig. 1A), was originally described from specimens from the Monterey Peninsula, California, USA, collected by the surgeon and naturalist Archibald Menzies while serving on the Vancouver Expedition from 1791 to 1795 (1–3). The species is a monocarpic perennial distributed from southern Oregon to central California, where it grows on coastal dunes, headlands, and cliffs (4). In 1992, due to declining population and environmental threats, such as residential development, competition from alien plants, and trampling by hikers, *E. menziesii* was placed on the federally endangered species list (5). The latest census in 2017 found 2,206 individual plants (6). Numerous complete *Erysimum* chloroplast genomes have been published to date (7–10); however, Menzies’ wallflower has not been sequenced. In this study, we assembled and documented the complete chloroplast genome of *E. menziesii* to contribute to the bioinformatics and conservation genetics of this extremely rare species.

The specimen of *E. menziesii* was collected with incidental take permit number 2081(a)−23-097-V from Asilomar, Pacific Grove, California (36°37′16.7″ N 121°56′22.8″ W) and deposited in the Humboldt State University Vascular Plant Herbarium (HSC), voucher number HSC 106351. The DNA was extracted from silica gel dried leaf material using the DNeasy Blood and Tissue Kit (Qiagen) following the manufacturer’s protocol with two modifications: the binding step was 4,000 × *g* for 3 minutes, and the DNA was eluted in 40 µL TAE after 7 minutes of incubation (12). The 150 bp paired-end library was constructed with the NEBNext Ultra II DNA Library Prep kit (New England Biolabs) and sequenced with RTA3 quality control software on an Illumina NovaSeq 6000 (Illumina, Inc.) by Novogene Corporation Inc. The sequencing generated 21,122,994 reads that were filtered using the default BBDuk 1.0 (13) settings in Geneious Prime 2019.1.3 (Biomatters Limited). The chloroplast genome was assembled using the filtered reads and the seed sequence, *E. siliculosum* (GenBank accession number JN847822) with default parameters in NOVOPlasty 4.3.1 (14). The assembler recovered a single, circular, chloroplast genome with an average coverage of 1,532×. The genome start position was manually rearranged to correspond with *E. cheiranthoides* (GenBank accession number MN207123). The annotation was predicted in default mode with CPGAVAS2 (15) using *E. cheiranthoides* as a reference and manually adjusting start and stop positions using default settings according to NCBI ORFfinder (https://www.ncbi.nlm.nih.gov/orffinder/), tRNAscan-SE 2.0 (16), and Sequin 15.5 (17).

The complete circular chloroplast genome of *E. menziesii* is 154,668 bp in length and displays a characteristic flowering plant quadripartite structure (18), containing a large 83,863 bp single-copy region, 17,847 bp small single-copy region, and two 26,479 bp inverted repeats (Fig. 1B). Gene content, organization, AT% (63.4%), and length are consistent with other published chloroplast genomes of *Erysimum* spp. (7–10). The *E. menziesii* genome has 130 genes, including 85 protein-coding, 37 tRNA, 8 rRNA genes (Fig. 1B). Four ribosomal genes (*rrn4.5S*, *rrn5S*, *rrn16S*, and *rrn23S*) and seven protein-coding genes (*ndhB*, *rpl2*, *rpl23*, *rps7*, *rps12*, *ycf1*, and *ycf2*) occur in duplicate (Table 1). Twelve of the tRNAs occur singly, four in duplicate, three in triplicate, and two in quadruplicate (Table 1).

**TABLE 1:** Chloroplast genome content of *Erysimum menziesii* from Asilomar, California, USA

Gene categories	Genes
Acetyl-CoA-carboxylase	accD
ATP synthase	atpA, atpB, atpE, atpF, atpH, atpI
C-type cytochrome synthesis gene	ccsA
Cytochrome b/f complex	petA, petB, petD, petG, petL, petN
DNA-dependent RNA polymerase	rpoA, rpoB, rpoC1, rpoC2
Envelope membrane protein	cemA
Hypothetical proteins	ycf1 (×2), ycf2 (×2), ycf4
Large ribosome subunits	rpl2 (×2), rpl14, rpl16, rpl20, rpl22, rpl23 (×2), rpl32, rpl33, rpl36
Maturase	matK
NADH-dehydrogenase	ndhA, ndhB, ndhB (×2), ndhC, ndhD, ndhE, ndhF, ndhG, ndhH, ndhI, ndhJ, ndhK
Photosystem I	psaA, psaB, psaC, psaI, psaJ
Photosystem II	psbA, psbB, psbC, psbD, psbE, psbF, psbI, psbJ, psbK, psbL, psbM, psbN, psbT, psbZ, ycf3
Protease	clpP
Ribosomal RNAs	rrn4.5S (×2), rrn5S (×2), rrn16S (×2), rrn23S (×2)
Rubisco	*rbc*L
Small subunit of ribosome	rps2, rps3, rps4, rps7 (×2), rps8, rps11, rps12 (×2), rps14, rps15, rps16, rps18, rps19
Transfer RNAs	trnA (×2), trnC, trnD, trnE, trnF, trnG (×2), trnH, trnI (×4), trnK, trnL (×4), trnM, trnfM, trnN (×2), trnP, trnQ, trnR (×3), trnS (×3), trnT (×2), trnV (×3), trnW, trnY

## Data Availability

The complete chloroplast genome sequence of *Erysimum menziesii* is available in GenBank under accession number PX508916. The associated BioProject, SRA, and BioSample numbers are PRJNA1357891, SRR35954535, and SAMN53093995, respectively.
